# Supplementary material for: Degradation of Poliovirus Sabin 2 Genome After Electron Beam Irradiation
Source: Vaccines (Basel). 2025 Jul 31;13(8):824. doi: 10.3390/vaccines13080824 (PMC12390167; doi:10.3390/vaccines13080824)
Supplement: Supplementary file 1 [file vaccines-13-00824-s001.zip › vaccines-3720091-supplementary.pdf]

Degradation of Poliovirus Sabin 2 Genome After Electron Beam Irradiation

Dmitry D. Zhdanov [1,2,*], Anastasia N. Shishparenok [1], Yury Y. Ivin [1,3], Anastasia A. Kovpak [3], Anastasia N. Piniaeva [1,3], Igor V. Levin [3], Sergei V. Budnik [4], Oleg A. Shilov [4], Roman S. Churyukin [4], Lubov E. Agafonova [1], Alina V. Berezhnova [1], Victoria V. Shumyantseva [1,5] and Aydar A. Ishmukhametov [3]

[1] Institute of Biomedical Chemistry, 10 Pogodinskaya str., 119121 Moscow, Russia; a.shishparyonok@yandex.ru (A.N.S.); ivin_uu@chumakovs.su (Y.Y.I.); pinyaeva_an@chumakovs.su (A.N.P.); agafonovaluba@mail.ru (L.E.A.); alinalikeed@mail.ru (A.V.B.); viktoria.shumyantseva@ibmc.msk.ru (V.V.S.)

[2] Department of Biochemistry, People's Friendship University of Russia Named After Patrice Lumumba (RUDN University), 6 Miklukho-Maklaya str., 117198 Moscow, Russia

[3] Chumakov Federal Scientific Center for Research and Development of Immune-and-Biological Products of Russian Academy of Sciences, 8/1 Polio Institute Settlement, Moskovsky Settlement, 108819 Moscow, Russia; kovpak_aa@chumakovs.su (A.A.K.); levin_iv@chumakovs.su (I.V.L.); ishmukhametov@chumakovs.su (A.A.I.)

[4] Teocortex LLC., 34/6 Pervomaysky Settlement, 108808 Moscow, Russia; sbudnik@teocortex.com (S.V.B.); oshilov@teocortex.com (O.A.S.); rchuryukin@teocortex.com (R.S.C.)

[5] Department of Biochemistry, Pirogov Russian National Research Medical University, 1 Ostrovityanova str., 117997 Moscow, Russia

* Correspondence: zhdanovdd@gmail.com

Supplementary File

Table S1. Segmenting of poliovirus Sabin 2 genomic RNA for real-time RT-PCR purposes

Region Name	Region Description	Sequence (5'-3')
CloSpa	Cloverleaf+Spacer	TTAAAACAGCTCTGGGGTTGTACCCACCCCAGAGGCCCACGTGGCGGCTAGCACTC<br>CGGTATTACGGTACCCTTGTGCGCCTGTTTTATACTCCCCTCCCGCAACTTAGAAG<br>CACGAAACCA
IRES	Internal ribosome entry site (IRES)	AGTTCAATAGAAGGGGGTACAAACCAGTACCACTACGAACAAGCACTTCTGTTTCC<br>CCGGTGACATTGCATAGACTGCTCACGCGGTTGAAAGTGATCGATCCGTTACCCGC<br>TTGTGTACTTCGAAAAGCCTAGTATCGCCTTGGAATCTTCGACGCGTTGCGCTCAG<br>CACCCGACCCCGGGGTGTAGCTTAGGCTGATGAGTCTGGACATTCCTCACCGGTGA<br>CGGTGGTCCAGGCTGCGTTGGCGGCCTACCTATGGCTAACGCCATAGGACGTTAGA<br>TGTGAACAAGGTGTGAAGAGCCTATTGAGCTACATAAGAGTCCTCCGGCCCCTGAA<br>TGCGGCTAATCCTAACCACGGAACAGGCGGTCGCGAACCAGTGACTGGCTTGTCGT<br>AACGCGCAAGTCTGTGGCGGAACCGACTACTTTGGGTGTCCGTGTTTCCTGTTATT<br>TTTATCATGGCTGCTTATGGTGACAATCAGAGATTGTTATCATAAAGCGA
VP4	Capsid protein VP4	ATGGGCGCCCAAGTTTCATCACAGAAAGTTGGAGCCCACGAAAATTCAAACAGAGC<br>CTATGGCGGGTCCACCATCAATTACACTACAATCAATTACTATAGGGACTCTGCAA<br>GCAATGCAGCAAGCAAGCAAGATTTTGCACAAGATCCGTCCAAGTTCACCGAACCC<br>ATTAAGGACGTCCTTATTAAGACCGCTCCCATGCTAAAC
VP2 left	Capsid protein VP2 (left part)	TCCCCAAACATTGAGGCGTGTGGTTATAGTGACAGGGTAATGCAGCTAACTCTGGG<br>CAATTCAACGATCACCACCCAAGAAGCGGCCAATTCTGTTGTTGCCTACGGTAGAT<br>GGCCTGAATACATCAGAGATACCGAGGCAAATCCTGTAGACCAACCAACCGAGCCC<br>GATGTAGCCGCGTGCAGGTTCTACACATTAGATACCGTCACTTGGCGCAAGGAGTC<br>CAGAGGGTGGTGGTGGAAACTACCAGACGCTTTAAAAGACATGGGGTTATTTGGTC<br>AAAACATGTTTTATCACTATCTTGGGAGGGCTGGCTACACAGTGCACGTACAGTGC

		AATGCTTCAAAGTTTCATCAAGGAGCTCTAGGGGTGTTTGCAGTTCCAGAAATGTG<br>TTTAGCTGGTGATAG
VP2 right	Capsid protein VP2 (right part)	CACAACTCACATGTTCACAAAGTACGAGAATGCGAATCCAGGCGAAAAAGGAGGTG<br>AATTCAAAGGGAGTTTCACCCTTGATACCAACGCCACTAACCCTGCACGGAACTTC<br>TGCCCAGTTGATTACCTCTTCGGGAGTGGAGTGCTGGTAGGGAATGCATTTGTTTA<br>TCCACATCAAATAATAAACCTGCGCACTAACAACTGTGCTACGCTAGTATTGCCCT<br>ATGTAAACTCACTCTCAATAGATAGCATGACAAAGCACAACAACTGGGGGATCGCT<br>ATCCTCCCCCTGGCGCCACTAGACTTTGCCACTGAATCTTCCACTGAGATACCCAT<br>TACACTGACCATTGCTCCCATGTGCTGCGAATTCAATGGTTTACGCAACATCACTG<br>TGCCAAGAACCCAA
VP3 left	Capsid protein VP3 (left part)	GGATTACCAGTCCTGAACACTCCAGGGAGTAACCAGTACCTGACCGCAGACAATTA<br>CCAGTCTCCGTGTGCGATACCTGAGTTTGATGTCACTCCACCCATAGACATACCAG<br>GGGAGGTGCGCAACATGATGGAATTGGCGGAAATAGACACCATGATACCCCTCAAC<br>TTGACAAGTCAACGCAAGAACACAATGGACATGTATAGAGTCGAGTTGAGCGACAC<br>GGCTCACTCTGACACGCCGATCTTGTGTCTCTCGTTGTCCCCCGCTTCAGACCCCA<br>GATTGGCACACACTATGTTGGGTGAGATATTAAATTACTACACACACTGGGCAGGG<br>TCCTTGAAATTTACCTTTCTC
VP3 right	Capsid protein VP3 (right part)	TTTTGCGGCTCAATGATGGCCACCGGAAAGTTATTGGTTTCTTACG<br>CACCACCCGGAGCAGAGGCCCCCAAGAGTCGCAAAGAAGCAATGCTTGGGACACAT<br>GTGATATGGGACATTGGGTTGCAGTCTTCATGCACTATGGTGGTACCTTGGATCAG<br>TAATACCACATACAGACAAACCATCAACGATAGTTTCACAGAAGGTGGCTACATTA<br>GCATGTTCTATCAAACTAGGGTTGTTGTCCCGTTGTCCACACCCAGAAAGATGGAC<br>ATCCTGGGTTTTGTGTCAGCTTGCAATGACTTCAGTGTGCGCTTACTGCGAGATAC<br>AACACACATTAGTCAAGAGGCTATGCCACAA
VP1 left	Capsid protein VP1 (left part)	GGAATTGGTGACATGATTGAGGGGGCCGTTGAAGGGATTACTAAAAATGCATTGGT<br>TCCCCCGACTTCCACCAATAGCCTGCCTGACACAAAGCCGAGCGGTCCAGCCCACT<br>CCAAGGAGATACCTGCATTGACAGCCGTGGAGACAGGGGCTACCAATCCGTTGGTG<br>CCTTCGGACACCGTGCAAACGCGCCATGTCATCCAGAGACGAACGCGATCAGAGTC<br>CACGGTTGAGTCATTCTTTGCAAGAGGGGCTTGCGTGGCTATCATTGAGGTGGACA<br>ATGATGCACCGACAAAGCGCG
VP1 middle	Capsid protein VP1 (middle part	CCAGCAGATTGTTTTCGGTTTGGAAAATAACTTACAAAGATACTGTTCAACTGAGA<br>CGCAAACTGGAATTTTTCACATATTCGAGATTTGACATGGAGTTCACTTTTGTGGT<br>CACCTCAAACTACATTGATGCAAATAACGGACATGCATTGAACCAAGTTTATCAGA<br>TAATGTATATACCACCCGGAGCACCTATCCCTGGTAAATGGAATGACTATACGTGG<br>CAGACGTCCTCTAACCCGTCGGTGTTTTACACCTATGGGGCGCCCCCAGCAAGAAT<br>ATCAGTGCCCTACGTGGGAAT
VP1 right	Capsid protein VP1 (right part)	TGCTAATGCGTATTCCCACTTTTATGATGGGTTTGCAAAAGTACCACTAGCGGGTC<br>AAGCCTCAACTGAAGGCGATTCGTTGTACGGTGCTGCCTCACTGAATGATTTTGGA<br>TCACTGGCTGTTCGCGTGGTAAATGATCACAACCCCACGCGGCTCACCTCCAAGAT<br>CAGAGTGTACATGAAGCCAAAGCATGTCAGAGTCTGGTGCCCACGACCTCCACGAG<br>CAGTCCCATACTTCGGACCAGGTGTTGATTATAAAGATGGGCTCACCCCACTACCA<br>GAAAAGGGATTAACGACTTAT
2A	Protease 2A	GGATTTGGACACCAAAACAAAGCTGTGTACACAGCTGGCTACAAAATTTGCAATTA<br>CCACCTAGCTACACAAGAAGACTTGCAAAATGCCGTGAGTGTCATGTGGAACAGAG<br>ACCTCTTAGTGGCTGAATCAAGGGCCCTTGGCACCGACTCGATCGCAAGGTGCAGC<br>TGTAACACGGGTGTGTACTACTGTGAATCCAGGAGAAAATATTATCCAGTTTCTTT<br>CATTGGGCCCACCTTCCAATACATGGAAGCCAATGAATATTACCCGGCTAGATATC<br>AATCACACATGCTTATTGGTCATGGGTTTGCATCACCGGGTGATTGTGGTGGCATA<br>CTTAGATGTCAACACGGGGTGATAGGAATAATCACTGCTGGTGGGAAGGCTTGGT<br>TGCATTTTCAGACATTAGAGACCTGTATGCTTATGAGGAGGAAGCTATGGAGCAG
2B	Protein 2B	GGCATTTCCAACTATATTGAGTCACTTGGTGCTGCATTTGGTAGTGGATTCACTCA<br>ACAAATTGGTGATAAAGTTTCCGAGCTAACCAGCATGGTAACTAGCACCATTACAG<br>AGAAGTTGCTTAAAAACTTAATCAAAATTATCTCATCACTTGTGATCATTACCAGG<br>AATTATGAGGACACTACCACAGTGCTTGCCACCCTCGCCCTCCTTGGGTGCGACAT<br>CTCACCGTGGCAGTGGCTAAAGAAGAAGGCATGTGACATCCTGGAAATTCCATACG<br>CCATCAAACAA
2C left	Protein 2C (left part)	GGAGATAGTTGGTTGAAGAAATTCACTGAGGCATGTAATGCTGCAAAGGGACTGGA<br>GTGGGTGTCCAATAAGATATCCAAATTCATTAGTTGGTTGCAGGATAAAATCATCC<br>CACAAGCGAGAGACAAATTAGAGTTTGTCACTAAACTAAAGCAATTAGAAATGCTT<br>GAAAATCAGATTTCCACCATACACCAATCTTGTCCAAGTCAAGAACATCAGGAGAT<br>CTTATTCACAATGTGCGGTGGCTATCTATCCAGTCCAAGAGGTTTGCACCACTATA<br>TGCACATGAAGCTAAAAGGATTCAAAAGCTGGAGCATACCATAAATAA

2C middle	Protein 2C (middle part)	TTACGTACAGTTCAAGAGCAAGCACCGTATTGAGCCAGTATGTTTGTTAGTACATG<br>GCAGTCCAGGGACAGGAAAATCAGTTGCAACCAATCTAATTGCTAGAGCAATAGCC<br>GAGAAAGAGAACACCTCCACATACTCACTGCCACCTGATCCGTCTCACTTTGATGG<br>CTACAAGCAACAGGGTGTGGTTATTATGGATGACCTAAACCAAAATCCAGACGGAG<br>CAGACATGAAACTTTTTTGTCAAATGGTGTCCACTGTGGAGTTTATTCCACCGATG<br>GCCTCGCTAGAAGAGAAAGGCATTTTGTTCACATCTAATTACGTTTTA
2C right	Protein 2C (right part)	GCCTCCACCAACTCCAGTCGGATCACACCACCCACGGTGGCTCACAGTGAT<br>GCGCTGGCCAGGAGATTCGCATTTGACGTGGACATACAAGTCATGAGCGAGTACTC<br>CAGAGACGGAAAGCTCAACATGGCAATGGCTACTGAAATGTGCAAAAACTGTCATC<br>AACCAGCAAACTTCAAAAGATGTTGTCCTTTAGTGTGTGGCAAGGCAATTCAGTTA<br>ATGGATAAATCTTCCAGGGTTAGATACAGCATTGATCAGATCACTACAATGATTGT<br>TAATGAGAGAAACAGAAGATCAAACATTGGTAATTGCATGGAAGCTCTATTCCAG
3A	Protein 3AB	GGACCACTGCAGTATAAAGATCTAAAAATAGATGTTAAGACCAGTCCCCCTCCGGA<br>GTGTATCAACGATTTGCTCCAGGCAGTTGATTCCCAGGAAGTGAGAGATTACTGTG<br>AAAAGAAAGGCTGGATTGTTAACATTACCAGTCAGGTTCAAACAGAGAGGAACATC<br>AACCGGGCGATGACTATCCTACAAGCAGTAACTACTTTCGCTGCAGTAGCCGGTGT<br>CGTGTACGTTATGTACAAGCTGTTCGCTGGGCACCAG
3C	Protease 3C	GGTGCATACACTGGTTTGCCAAATAAACGACCCAATGTACCCACTATCAGGACAGC<br>AAAAGTGCAAGGCCCTGGGTTTGATTACGCAGTGGCCATGGCTAAAAGAAACATTG<br>TTACAGCAACCACCAGCAAAGGGGAGTTTACGATGTTGGGAGTCTATGATAATGTG<br>GCCATCTTGCCAACCCACGCCTCACCTGGTGAAAGCATTGCGATCGACGGTAAAGA<br>GGTGGAAATTCTTGACGCCAAAGCCCTTGAAGATCAGGCAGGAACTAATCTTGAAA<br>TTACCATAATTACACTAAAGAGGAACGAGAAGTTCAGAGATATCAGGCCACACATT<br>CCCACCCAAATCACCGAAACAAATGATGGAGTTTTGATCGTGAACACTAGTAAGTA<br>CCCCAACATGTATGTTCCCGTTGGTGCTGTGACCGAACAGGGGTATCTTAATCTCG<br>GTGGACGACAAACCGCTCGTACGCTAATGTACAACTTTCCAACTAGAGCAGGTCAG<br>TGTGGTGGTGTCATCACGTGCACTGGTAAAGTCATTGGGATGCATGTTGGTGGGAA<br>CGGTTCACATGGGTTCGCGGCGGCCCTAAAGCGGTCATACTTCACTCAGATTCAA
3D left	3D protein (left part)	GGTGAGATTCAATGGATGAAACCATCAAAAGAAGTGGGATACCCGATCATAAATGC<br>TCCGTCCAAAACCAAACTTGAACCCAGCGCTTTTCACTATGTGTTTGAAGGGGTGA<br>AGGAACCAGCAGTCCTTACCAAAAATGATCCCAGGCTCAGGACAGACTTTGAAGAA<br>GCAATATTCTCTAAGTATGTAGGCAACAAGATCACTGATGTGGATGAGTACATGAA<br>AGAGGCAGTGGATCATTACGCTGGCCAACTCATGTCTCTAGACATCAACACAGAAC<br>AAATGTGCTTGGAGGACGCCATGTACGGCACCGATGGCCTGGAAGCACTTGACTTG<br>ACCACTAGTGCTGGATACCCTTATGTAGCAATGGGAAAGAAAAAGAGAGACATCTT<br>GAATAAGCAGACTAGAGACACCAAGGAAATGCGGAGACTCTTAGATACTTATGGAA<br>TTAACTTACCGCT
3D middle	3D protein (middle part)	TGTAACATATGTTAAAGATGAACTAAGGTCAAAAACTAAGGTGGAGCAGGGAAAAT<br>CCAGATTGATTGAAGCCTCCAGTTTGAATGATTCAGTGGCCATGAGAATGGCATTT<br>GGAAATCTCTATGCAGCATTTCACAAAAACCCAGGAGTTGTCACTGGCAGTGCAGT<br>TGGTTGTGATCCAGATCTATTTTGGAGCAAGATCCCAGTGCTAATGGAAGAGAAGC<br>TCTTTGCTTTTGACTACACAGGTTATGATGCATCACTCAGCCCGGCCTGGTTTGAG<br>GCACTCAAAATGGTGCTAGAGAAAATCGGATTTGGGGACAGGGTGGATTATATTGA<br>TTACCTCAACCATTCCCACCACCTGTACAAAAACAAAACTTATTGCGTAAAAGGCG<br>GCATGCCATCTGGCTGCTCAGGCACATCAATTTTTAACTCAATGATTAACAACTTA<br>ATCATTAGGACAC
3D right	3D protein (right part)	TCCTACTGAAAACCTACAAGGGCATAGATTTAGATCACCTAAAGATGATTGCCTAT<br>GGTGATGATGTAATTGCTTCCTACCCCCATGAGGTTGATGCTAGTCTCCTAGCCCA<br>ATCAGGAAAAGACTATGGACTAACCATGACTCCAGCAGACAAGTCAGCTACCTTTG<br>AAACAGTCACATGGGAGAATGTAACATTCTTGAAAAGATTCTTTAGAGCGGATGAG<br>AAGTATCCCTTCCTCATACATCCAGTAATGCCAATGAAGGAGATTCATGAATCAAT<br>TAGATGGACAAAGGATCCCAGAAACACACAGGATCACGTGCGCTCATTGTGCCTAT<br>TGGCCTGGCACAACGGCGAAGAAGAATACAACAAGTTCTTAGCTAAAATCAGGAGT<br>GTGCCAATTGGGAGAGCTTTATTGCTCCCAGAGTACTCTACATTGTACCGCCGTTG<br>GCTCGACTCTTTT
Stem loop	Stem loop	TAGTAACCCTACCTCAGTCGAATTGGATTGGGTCATGCTGTTGTAGGGGTAAATTT<br>TTCTTTAATTCGGGG

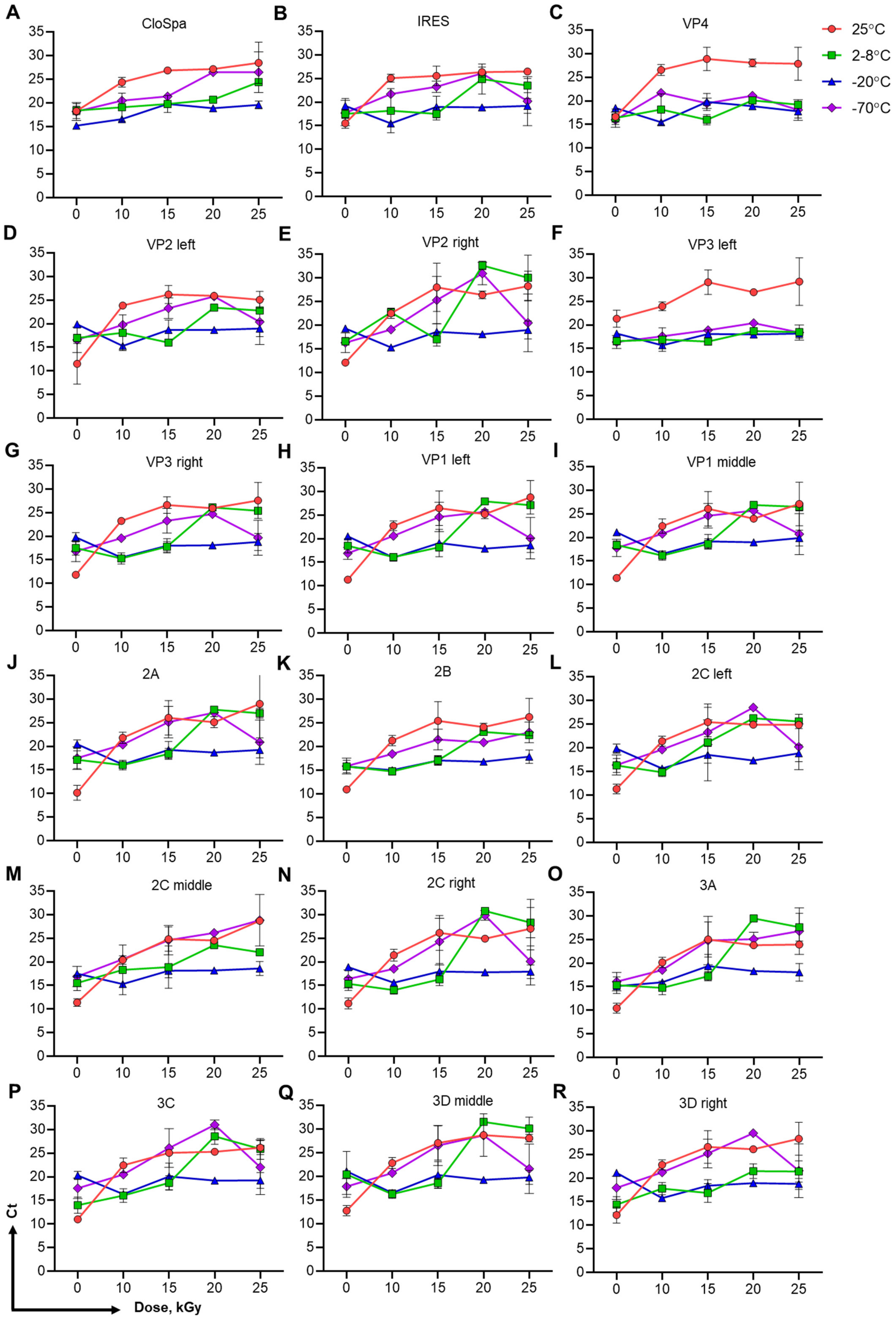

A
CloSpa
B
IRES
C
VP4
25°C
2-8°C
-20°C
-70°C
D
VP2 left
E
VP2 right
F
VP3 left
G
VP3 right
H
VP1 left
I
VP1 middle
J
2A
K
2B
L
2C left
M
2C middle
N
2C right
O
3A
P
3C
Q
3D middle
R
3D right
Ct
Dose, kGy

Figure S1. Plots of Ct versus irradiation dose for the Sabin 2 virus strain irradiated at different temperatures. Samples were irradiated with accelerated electrons in the range of 10-25 kGy (50 mEV). Total RNA was isolated and subjected to qPCR using primers specific for different sites of viral RNA. Viral RNA sites (A) CloSpa; (B) IRES; (C) VP4; (D) VP2 left; (E) VP2 right; (F) VP3 left; (G) VP3 right; (H) VP1 left; (I) VP1 middle; (J) 2A; (K) 2B; (L) 2C left; (M) 2C middle; (N) 2C right; (O) 3AB; (P) 3C; (Q) 3D middle; (R) 3D right.

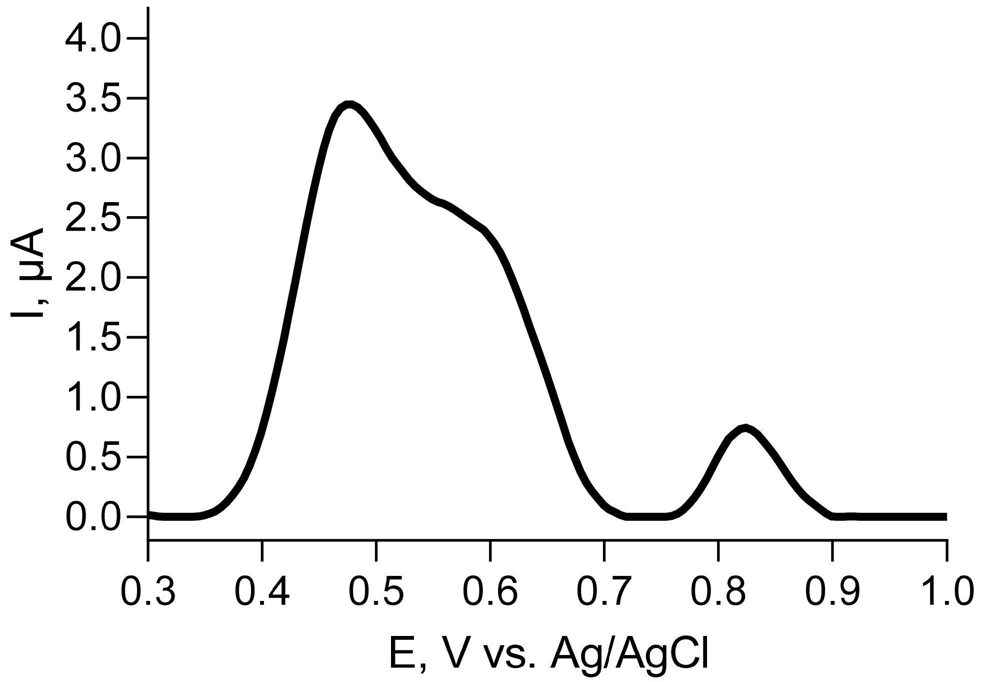

Figure S2. DPV fingerprint for SPE/SWCNT in the presence of the poliovirus Sabin 2 genome at a concentration of 402 µg/mL.
